# CODECHECK: an Open Science initiative for the independent execution of computations underlying research articles during peer review to improve reproducibility

**DOI:** 10.12688/f1000research.51738.2

**Published:** 2021-07-20

**Authors:** Daniel Nüst, Stephen J. Eglen

**Affiliations:** 1 Institute for Geoinformatics, University of Münster, Münster, Germany; 2 Department of Applied Mathematics and Theoretical Physics, University of Cambridge, Cambridge, UK

**Keywords:** reproducible research, Open Science, peer review, reproducibility, code sharing, data sharing, quality control, scholarly publishing

## Abstract

The traditional scientific paper falls short of effectively communicating computational research.  To help improve this situation, we propose a system by which the computational workflows underlying research articles are checked. The CODECHECK system uses open infrastructure and tools and can be integrated into review and publication processes in multiple ways. We describe these integrations along multiple dimensions (importance, who, openness, when). In collaboration with academic publishers and conferences, we demonstrate CODECHECK with 25 reproductions of diverse scientific publications. These CODECHECKs show that asking for reproducible workflows during a collaborative review can effectively improve executability. While CODECHECK has clear limitations, it may represent a building block in Open Science and publishing ecosystems for improving the reproducibility, appreciation, and, potentially, the quality of non-textual research artefacts. The CODECHECK website can be accessed here: https://codecheck.org.uk/.

## Abbreviations

ACM: Association for Computing Machinery; ECRs: Early Career Researchers; RCR: Replicated Computational Results; TOMS: Transactions on Mathematical Software.

## Introduction

**Figure 1.**
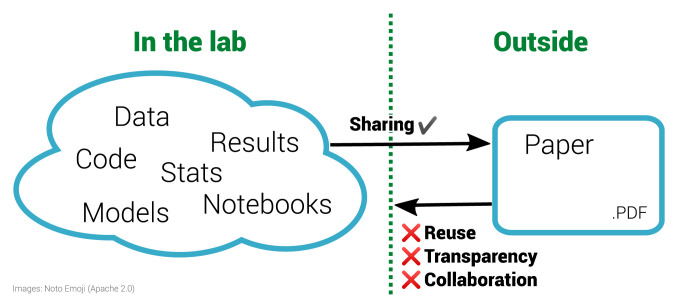
The inverse problem in reproducible research. The left half of the diagram shows a diverse range of materials used within a laboratory. These materials are often then condensed for sharing with the outside world via the research paper, a static PDF document. Working backwards from the PDF to the underlying materials is impossible. This prohibits reuse and is not only non-transparent for a specific paper but is also ineffective for science as a whole. By sharing the materials on the left, others outside the lab can enhance this work.

Many areas of scientific research use computations to simulate or analyse their data. These complex computations are difficult to explain coherently in a paper ^1^. To complement the traditional route of sharing research by writing papers, there is a growing demand to share the underlying artefacts, notably code and datasets, so that others can inspect, reproduce or expand that work (see Figure 1). Early proponents of this initiative were Buckheit and Donoho ^2, 3^, who noted: *“An article about computational science in a scientific publication is not the scholarship itself, it is merely **advertising** of the scholarship. The actual scholarship is the complete software development environment and the complete set of instructions which generated the figures.”*

If researchers start sharing more artefacts, how might these artefacts be examined to ensure that they do what they claim? For example, although scientific journals now require a data sharing statement that outlines what data the authors have (or will) share, journals implement this differently. On one hand, journals have been created to accept “data papers” (e.g., *Scientific Data*, *Earth System Science Data*, *Geoscience Data Journal*, *Biodiversity Data Journal*, *Journal of Open Psychology Data*, *Open Data Journal for Agricultural Research*, *Journal of Open Health Data*); these journals have established rigorous procedures by which data are validated according to standards in each field. On the other hand, many journals still allow authors to state “Data available upon reasonable request”. Authors, while possibly well intentioned at the time of writing the article, often cannot provide data when requested as data disappears over time ^4^.

Given that data are not routinely shared, what hope might there be for sharing computer programs? Both data and software are required to validate a computational analysis; data can be seen as inert whereas code requires an environment to be run in. This makes software harder to share. Our experience is that researchers offer several reasons for why code is not shared, e.g., “there is no documentation”, “I cannot maintain it”, or “I do not want to give away my code to competitors”. Our view is that sharing code, wherever possible, is good for the community and the individual ^5, 6^. Having code and data openly available, and archived, provides a valuable resource for others to learn from, even if the code is broken or lacks documentation. However, with a little effort, we believe that if an independent person can re-run the programs, this is worth documenting and that this reduces the barrier to evaluating non-text research materials. Just as data journals’ validations of data and all journals’ peer review provides a “baseline reassurance”, i.e., that a paper has been checked by someone with an understanding of the topic ^7^, the same baseline could be provided for the computational workflow underlying a paper. With this in mind, we have developed a set of principles and an example CODECHECK workflow that provides a pragmatic way of checking that a paper’s code works, i.e., it is reproducible following the Claerbout/Donoho/Peng terminology ^8^.

Here we offer a thorough description of a process and its variations to integrate a much-needed evaluation of computational reproducibility into peer review, and we demonstrate its feasibility by means of 25 reproductions across scientific disciplines. We call this system CODECHECK.

## What is a CODECHECK?

### CODECHECK workflow and people

**Figure 2.**
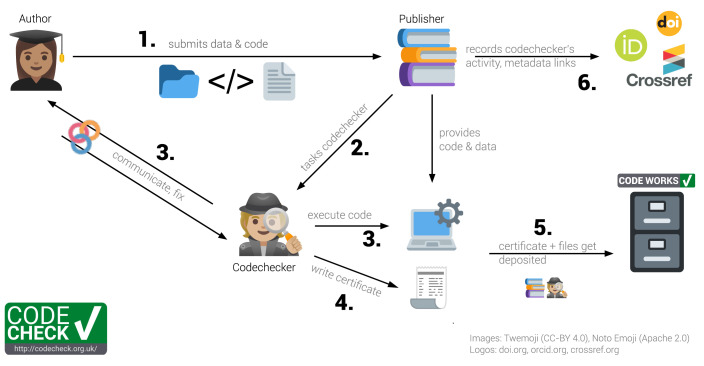
The CODECHECK example workflow implementation. Codecheckers act as detectives: They investigate and record, but do not fix issues. Numbers in bold refer to steps outlined in the text.

CODECHECK is best demonstrated by way of our example workflow, and later we expand on the underlying principles. The CODECHECK workflow involves three groups of people: (1) the **author** of a paper providing the code to be checked, (2) the **publisher** of a journal interested in publishing the author’s paper, and (3) the **codechecker**, who checks that the author’s code works. The six-step CODECHECK workflow we have refined is shown in Figure 2. In this article, we also refer to a **peer-reviewer** who is independent of this process, and performs the traditional academic review of the content of an article.

**Step 1:** The author submits their manuscript along with the code and data to the publisher. The code and data need not be openly available at this point. However, in many cases the code and data may be published on a code hosting platform, such as GitHub or GitLab. Ideally, the author is expecting the CODECHECK and prepares for it, e.g., by asking a colleague to attempt a reproduction, and providing a set of instructions on how to re-run the computational workflow.

**Step 2:** The publisher finds a codechecker to check the code. This is analogous to the publisher finding one or more peer-reviewers to evaluate the paper, except we suggest that the codechecker and the author talk directly to each other.

**Step 3:** The codechecker runs the code, based on instructions provided by the author. They check if some or all of the results from the paper can be reproduced. If there are any problems running the code, the codechecker asks the author for help, updates, or further documentation. The burden to provide reproducible material lies with the author. The codechecker then tries to run the code again. This process iterates until either the codechecker is successful, or the codechecker concludes the paper’s workflow is not reproducible. As part of this process, the codechecker could work entirely locally, relying on their own computing resources, or in the cloud, e.g., using the open MyBinder infrastructure ^9^ or alternatives, some of which are more tailored to scientific publications while others offer commercial options for, e.g., publishers (cf. 10). A cloud-based infrastructure allows for the codechecker and author to collaboratively improve the code and enforces a complete definition of the computing environment; but, unless secure infrastructure is provided, e.g., by the publisher, this requires the code and data to be published openly online. Note that the task of the codechecker is to check only the “mechanics” of the computational workflow. In the context of mathematics, Stodden *et al*. ^11^ distinguish between *verification* and *validation*; following their definition, a CODECHECK ensures verification of computational results, i.e., checking that code generates the output it claims to create, but not a validation, i.e., checking that the code implements the right algorithm to solve the specific research problem. Nevertheless, simply attempting to reproduce an output may highlight a submission’s shortcomings in meeting a journal’s requirements (cf. 12) and may effectively increase transparency, thereby improving practices (cf. 13) even if the check does not go into every detail.

**Step 4:** The codechecker writes a certificate stating how the code was run and includes a copy of outputs (figures or tables) that were independently generated. The certificate may include recommendations on how to improve the material. The free text in the certificate can describe exactly what was checked, because each computational workflow is unique. Since no specific tool or platform is required, such that no authors are excluded, it is futile for the codechecker to use automation or fixed checklists.

**Step 5:** The certificate and auxiliary files created during the check, e.g., a specification of a computing environment, data subsets or helper scripts, and the original code and data get deposited in an open archive unless restrictions (data size, license or sensitivity) apply. Currently, codecheckers deposit the material on Zenodo themselves, but a publisher may complete this step after integrating CODECHECK into its review process. A badge or other visual aid may be added to the deposit and the paper and link to the certificate. Although a badge simplifies the CODECHECK into a binary value and risks introducing confusion regarding the extent of the check, a badge provides recognition value and acknowledges the completed CODECHECK. The badge and the actual check are incentives for undertaking the effort needed to provide a reproducible workflow.

**Step 6:** The publisher can, depending on the timing, provide the certificate to peer-reviewers or editors or publish it and link between certificate, paper, and any repositories. Currently, the codechecker creates these connections on Zenodo. They appear as links with a relationship type on the Zenodo landing page for a certificate, e.g., the “related identifiers” and “alternate identifiers” of certificate 2020-025 ^14^. The publisher also credits the codechecker’s work by depositing the activity in scholarly profiles, such as ORCID (see peer review contributions in ORCID records). The publisher also ensures proper publication metadata, e.g., links from the certificate repository to the published paper or the original code repository.

### Variations

**Figure 3.**
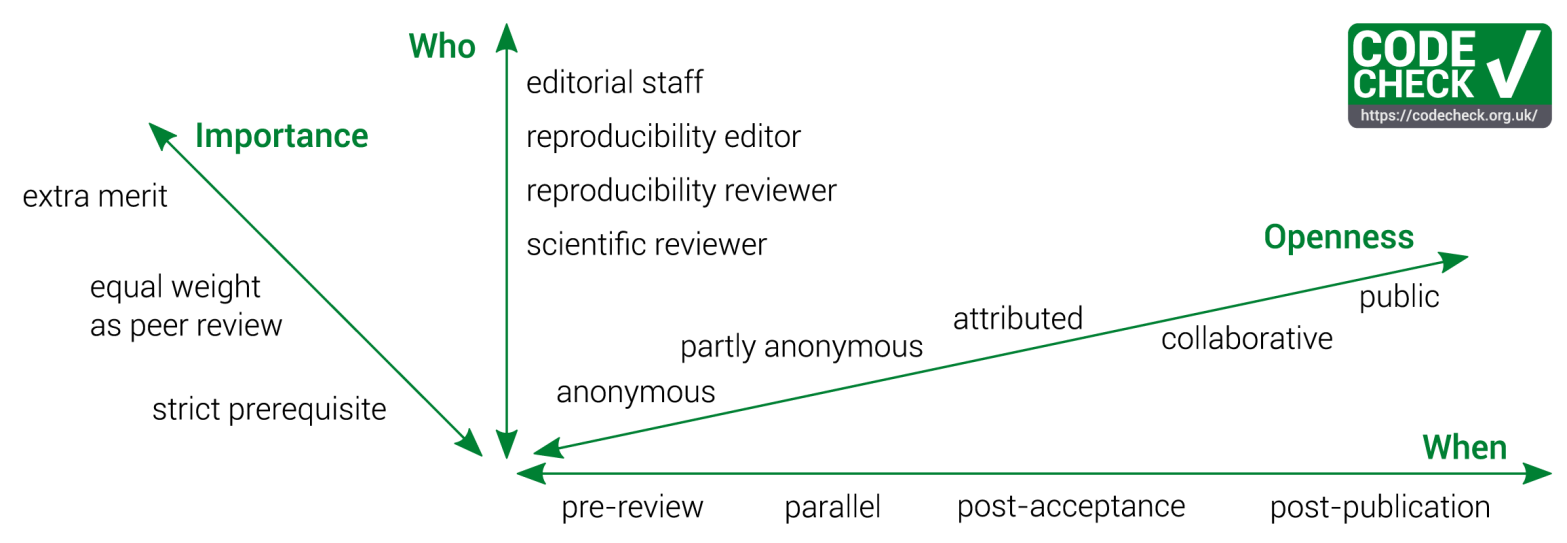
The dimensions of implementing a CODECHECK workflow.

***Dimensions of CODECHECK workflows.*** Our workflow is just one of many possibilities of a CODECHECK workflow. Here we consider several dimensions in a space of possible CODECHECK workflows ( Figure 3). These aspects touch on timing, responsibilities, and transparency.

***When to do a CODECHECK and with what importance?*** The time at which a CODECHECK is done and its ascribed importance are closely connected, so we describe the dimensions *When* and *Importance* together. The earlier a CODECHECK happens in the publishing process, the more it can affect editorial decisions: Is a paper published, sent back for revisions, or rejected? Even earlier checks, i.e., a CODECHECK of a preprint, may help to improve the computational workflow itself, even before a publisher is involved. As such, codechecking papers could be part of a preprint server’s policy or initiated by interested authors.

Publishers could introduce a CODECHECK as a **strict prerequisite**. As this can reduce the workload of reviewers, such a check should occur early in the review process. Yet, the later in the review process the check happens, the easier is it to allow bidirectional communication between the author and codechecker, e.g., because the author might already be notified of the paper’s acceptance and may be more willing to share materials online closer to the paper’s publication date. A **pre-review** CODECHECK means editors would send a submission for peer review only if it passes the check, or include the certificate in the submission package provided to peer-reviewers. Peer-reviewers may then judge the relevance of the computations for the results of the work.

A CODECHECK may also be conducted in **parallel** to the academic peer review. This puts less burden on the turnaround time for the CODECHECK, yet it only makes the outcomes available during the final consideration by the handling editor. The check could also be assigned after suggestion by a reviewer, which would remove the need for submissions to undergo a pre-review screening. However, soliciting such a “specialist review” is much less desirable than having a regular CODECHECK, thus avoiding the situation in which some submissions get special treatment. In both cases, the editor’s decision could be based both on CODECHECK and peer-review reports.

A **post-acceptance** CODECHECK would have the smallest impact on editorial decisions and may simply provide **extra merit** on top of the submission’s acceptance. This is the least impactful solution in which all material is still evaluated and the results of the check are properly acknowledged, because the check can be completed before publication of the paper. The GIScience checks (see below) falls into this category: by displaying a badge on the volume and article landing pages, the AGILE conference highlights articles whose reproducibility was confirmed. Similarly, in collaborations with journals, some GIScience articles were checked whilst authors worked on revisions.

A CODECHECK may also be conducted **post-publication**, though this requires an update to the article and article metadata to reference the check so that readers can find the CODECHECK. In general, publishers hesitate to make such revisions to published articles. We do not prefer this option as it has the least impact on current publishing practices and downplays the importance of reproducible workflows for ensuring good scientific practice.

Enhancing existing review and publication processes with CODECHECKs allows communities to gradually transition towards more open practices. When integrating a CODECHECK into existing review and publication processes, the *turnaround time* is crucial. Depending on when and who conducts the check, it might be done quickly or it might delay publication. We found that a CODECHECK generally takes 2–5 hours, with some outliers on the higher end. This time includes writing and publishing the certificate but excludes actual computation time, some of which took days. These efforts are comparable to the time needed to peer review a submission, which aligns with the efforts some volunteer codecheckers are willing to make. Currently, there is considerable amount of communicating about the CODECHECK workflow, especially regarding who publishes which document when, so that proper cross-referencing between paper and certificate is ensured via persistent identifiers. When integrated into a peer review platform, this handling of documents should become much more streamlined.

***Openness, or “Who knows who?”*** Anonymity is broadly discussed, especially in the push towards open peer review as part of the Open Science movement (cf. 15). Without taking a strong stance on this topic, our motivation behind CODECHECK for higher transparency and reproducibility does indeed favour a more open review process. However, anonymity can protect individuals ^16^, e.g., junior scientists. The negative effects of a signed review may be reduced if a CODECHECK is not relevant for a journal’s decision to accept or reject, but that is, of course, not desirable when the goal is higher transparency and reproducibility. Instead, CODECHECK is a technical process that should generally find fixable problems; it is not aimed at giving an opinion or identifying a faulty approach. If passing a CODECHECK becomes mandatory, full transparency may need revisiting as the relations between authors and codecheckers would fall under the same social and community challenges as open peer review (cf. 17).

The technical nature of the check and the challenge of providing sufficient documentation is why we see great benefits in bidirectional communication between author and codechecker. Instead of trying to fix problems or guess the next step, the codechecker can ask the author to rework the documentation or update code. Instead of struggling to provide perfect instructions and as a result possibly not sharing any code or data, the author can make a best effort to document sufficiently. Authors and readers can profit from a codecheckers’ experience and approach, as during the check they may create useful and instructive files, e.g., a machine-readable computing environment specification. While communication between author and codechecker may be anonymised via the publisher, it most likely only helps to protect the identity of the codechecker, because code is hard to anonymise. Therefore, the most effective and desirable situation for the stakeholders is to hold a open and collaborative CODECHECK. The contributions by the codechecker may even be integrated into the code of the paper’s workflow and be acknowledged as code commits. This way, proper credit can be given within the research software development community.

***Who does the CODECHECK?*** Just as with peer-reviewers, a potential codechecker should have the right skills and availability to do the work. Ideally, the codechecker has a matching code *and* domain expertise to the paper, although a well-documented analysis should be executable by any computationally-competent person. Naturally, the more prerequisite knowledge the codechecker has, the quicker they can understand the goals and mechanics of an analysis. From our experiences, the priority should be given to matching technical expertise first, as lacking knowledge in setting up a computing environment with a particular language or tool is much more of a problem than assessing the outcome, e.g., comparing created figures with the original, without an in-depth understanding of the domain. The depth of the check will mostly be driven by the time required and expertise of the checker, though in general, we expect a CODECHECK to consider reproducibility of the results above performance of the code. Codecheckers could be drawn from a regular pool of **peer-reviewers**, or from a special group of **reproducibility reviewers** via specific roles such as **reproducibility editors**, or **editorial staff** with a publisher. One codechecker is sufficient to verify the paper’s workflow since it is mostly a factual process. Code usually harbours systematic and repeatable mistakes and is thereby more reliable and auditable than processes controlled by humans ^18^, e.g., in a laboratory. If however publication of the paper depends on the CODECHECK, a second opinion may be required.

We also see a great opportunity to involve *early-career researchers* (ECRs) as codecheckers. ECRs arguably have a high interest in learning about new tools and technologies, to build up their own expertise. CODECHECK offers a way for ECRs to gain insights into new research and highlight the importance of reproduction. *ReScience X*, a journal devoted to reproduction and replication experiments ^19^, shares an interest in this combination. ECRs are also often familiar with new technologies, thus also making them likely to author CODECHECK-ready manuscripts. A supporting data point for ECRs as early adopters is that they are responsible for 77% of 141 registered reports that were submitted ^20^. As ECRs are introduced to peer review as codecheckers, they may transition into the role of peer-reviewer over time. Overall, we see several opportunities and benefits to setting up a new process for codechecking with a clear commitment to openness and transparency, independent of the current peer review process (see *Openness* dimension).

The codechecker could be a member of **editorial staff**; this is the most controlled but also resource-intensive option. Such a resource commitment would show that publishers are investing in reproducibility, yet this commitment may be hard for small publishers. These codecheckers could be fully integrated into the internal publication process. Credit for doing the codecheck is also achieved, as it is part of their duties. By contrast, it is useful for researchers to be publicly credited for their reviewing activity. A regular review may be listed in public databases (e.g., ORCID, see Step 6 above, or commercial offerings such as Publons, and ReviewerCredits); a codechecker could be similarly listed. The codechecker community has over 20 volunteers who signed up in the last year, see https://github.com/codecheckers/codecheckers/. Their motivations, mentioned in the registration information, include: supporting reproducible research and Open Science, improve coding skills, gaining experience in helping scientists with their code, encouraging a sharing culture, and learning from other people’s mistakes; many are also motivated simply by curiosity. We see benefits to an open shared list of codecheckers across journals rather than a private in-house group, as this may allow for better matches regarding expertise and workload sharing. This community can establish CODECHECK as a viable option for independent no-cost Open Access journals.

### Core principles

The CODECHECK workflow and variations outlined describe our current views on how code could be checked. They are not immutable, but we believe the following core principles underpin our CODECHECK workflow:

**1. Codecheckers record but don’t investigate or fix.**

The codechecker follows the author’s instructions to run the code. If instructions are unclear, or if code does not run, the codechecker tells the author. We believe that the job of the codechecker is not to fix these problems but simply to report them to the author and await a fix. The level of documentation required for third parties to reproduce a computational workflow is hard to get right, and too often this uncertainty leads researchers to give up and not document it at all. The conversation with a codechecker fixes this problem.

**2. Communication between humans is key.**

Some code may work without any interaction, e.g. 21, but often there are hidden dependencies that need adjusting for a particular system. Allowing the codechecker to communicate directly and openly with the author make this process as constructive as possible; routing this conversation (possibly anonymously) through a publisher would introduce delays and inhibit community building.

**3. Credit is given to codecheckers.**

The value of performing a CODECHECK is comparable to that of a peer review, and it may require a similar amount of time. Therefore, the codechecker’s activity should be recorded, ideally in the published paper. The public record can be realised by publishing the certificate in a citable form (i.e., with a DOI), by listing codecheckers on the journal’s website or, ideally, by publishing the checks alongside peer review activities in public databases.

**4. Computational workflows must be auditable.**

The codechecker should have sufficient material to validate the computational workflow outputs submitted by the authors. Stark ^22^ calls this “preproducibility” and the ICERM report ^11^ defines the level “Auditable Research” similarly. Communities can establish their own good practices or adapt generic concepts and practical tools, such as publishing all building blocks of science in a research compendium (cf. https://research-compendium.science/) or “repro-pack” ^23^. A completed check means that code could be executed at least once using the provided instructions, and, therefore, all code and data was given and could be investigated more deeply or extended in the future. Ideally, this is a “one click” step, but achieving this requires particular skills and a sufficient level of documentation for third parties. Furthermore, automation may lead to people gaming the system or reliance on technology, which can often hide important details. All such aspects can reduce the understandability of the material, so we estimate our approach to codechecking, done without automation and with open human communication, to be a simple way to ensure long-term transparency and usefulness. We acknowledge that others have argued in favour of bitwise reproducibility because, in the long run, it can help to automate checking by comparing outputs algorithmically (e.g., https://twitter.com/khinsen/status/1242842759733665799), but until such an ideal is achievable we need CODECHECK’s approach.

**5. Open by default and transitional by disposition.**

Unless there are strong reasons to the contrary (e.g., sensitive data on human subjects), all code and data, both from author and codechecker, will be made freely available when the certificate is published. Openness is not required for the paper itself, to accommodate journals in their transition to Open Access models. The code and data publication should follow community good practices. Ultimately we may find that CODECHECK activities are subsumed within peer review.

## Implementation

### Register

To date we have created 25 certificates ( Table 1) falling into three broad themes: (1) classic and current papers from computational neuroscience, (2) COVID-19 modelling preprints, and (3) GIScience. The first theme was an initial set of papers used to explore the concept of CODECHECK. The idea was to take well-known articles from a domain of interest (Neuroscience). Our first CODECHECK (certificate number 2020-001) was performed before publication on an article for the journal *GigaScience*, which visusalized the outputs from a family of supervised classification algorithms.

**Table 1.** Register of completed certificates as of December 2020. An interactive version is available at http://codecheck.org.uk/register .

Certificate	Research area	Description
2020-001 ^30^	Machine learning	Code for benchmarking ML classification tool checked post acceptance of manuscript and before its publication in *Gigascience* ^31^.
2020-002 ^32^	Neuroscience	Code written for this project checked by second project member as demonstration using paper from 1997 showing unsupervised learning from natural images ^33^.
2020-003 ^34^	Neuroscience	Code written for this project checked by second project member as demonstration using classic paper on models of associative memory ^35^.
2020-004 ^36^	Neuroscience	Code written for this project checked by second project member as demonstration using classic paper on cart-pole balancing problem ^37^.
2020-005 ^38^	Neuroscience	Check of independent reimplementation of spike-timing-dependent plasticity (STDP) model ^39^ conducted as demonstration for this paper.
2020-006 ^40^	Neuroscience	Check of independent reimplementation of a generalized linear integrate-and-fire neural model ^41^ conducted as demonstration for this paper
2020-007 ^42^	Neuroscience	Check of independent reimplementation of analysing spike patterns of neurons ^43^ conducted as demonstration for this paper.
2020-008 ^44^	COVID-19	Code for modelling of interventions on COVID-19 cases in the UK checked at preprint stage ^45^ and later published ^24^.
2020-009 ^46^	COVID-19	Code for analysis of effectiveness of measures to reduce transmission of SARS-CoV-2 checked as preprint ^47^ and later published ^25^.
2020-010 ^27^	COVID-19	Code for analysis of non-pharmaceutical interventions (Report 9) checked as a preprint ^48^.
2020-011 ^49^	COVID-19	Code for modelling of COVID-19 spread across Europe was provided by authors and checked while paper was in press ^50^.
2020-012 ^51^	COVID-19	Code for modelling of COVID-19 spread across the USA was checked as preprint ^52^ and later published ^53^.
2020-013 ^21^	Neuroscience	Code for analysis of rest-activity patterns in people without con-mediated vision was checked as a preprint ^54^ after direct contact with the authors.
2020-014 ^55^	Neuroscience	Code for analysis of perturbation patterns of neural activity was checked after publication as part of publisher collaboration ^56^.
2020-015 ^57^	Neuroscience	Code for a neural network model for human focal seizures was checked after publication as part of publisher collaboration ^58^
2020-016 ^59^	GIScience	Code for models demonstrating the Modifiable Aral Unit Problem (MAUP) in spatial data science ^60^ was checked during peer review.
2020-017 ^61^	GIScience	Code for spatial data handling, analysis, and visualisation using a variety of R packages ^62^ was checked after peer review before publication.
2020-018 ^63^	GIScience	AGILE conference reproducibility report using a demonstration data subset with cellular automaton for modeling dynamic phenomena ^64^.
2020-019 ^65^	GIScience	AGILE conference reproducibility report with subsampled dataset for reachability analysis of suburban transportation using shared cars ^66^.
2020-020 ^67^	GIScience	AGILE conference reproducibility report using a container for checking in-database windows operators for processing spatio-temporal data ^68^.
2020-021 ^69^	GIScience	AGILE conference reproducibility report checking code for comparing supervised machine learning models for spatial nominal entity recognition ^70^.
2020-022 ^71^	GIScience	AGILE conference reproducibility report checking code for visualising text analysis on intents and concepts from geo-analytic questions ^72^.
2020-023 ^73^	GIScience	AGILE conference reproducibility report on analysis of spatial footprints of geo-tagged extreme weather events from social media ^74^.
2020-024 ^75^	Neuroscience	Code for multi-agent system for concept drift detection in electromyography ^76^ was checked during peer review.
2020-025 ^14^	GIScience	Adaptation and application of Local Indicators for Categorical Data (LICD) to archaeological data ^77^ was checked after peer review before publication.

The second theme was a response to the COVID-19 pandemic, selecting papers that predicted outcomes. The checks were solicited through community interaction or by our initiative rather than requested from journals. Some certificates were since acknowledged in the accepted papers ^24, 25^. In particular, we codechecked the well-known Imperial college model of UK lockdown procedures from March 2020, demonstrating that the model results were reproducible ^26, 27^.

The third theme represents co-author DN’s service as a Reproducibility Reviewer at the AGILE conference series, where the *Reproducible AGILE* Initiative ^28^ independently established a process for reproducing computational workflows at the AGILE conference series ^29^. While using slightly different terms and infrastructure (“reproducibility reports” are published on the Open Science Framework instead of certificates on Zenodo) AGILE reproducibility reviews adhere to CODECHECK principles. A few checks were also completed as part of peer reviews for GIScience journals.

### Annotated certificate and check metadata

After running the paper’s workflow, the codechecker writes a certificate stating which outputs from the original article, i.e., numbers, figures or tables, could be reproduced. This certificate is made openly available so that everyone can see which elements were reproduced and what limitations or issues were found. The certificate links to code and data used by the codechecker, allowing others to build on the work. The format of the certificates evolved during the project, as we learnt to automate different aspects of the certification. The metadata is stored in a machine-readable structured file in YAML, the CODECHECK configuration file codecheck.yml. The technical specification of the CODECHECK configuration file is published at https://codecheck.org.uk/spec/config/latest/. The configuration file enables current and future automation of CODECHECK workflows and meta-analyses.

**Figure 4.**
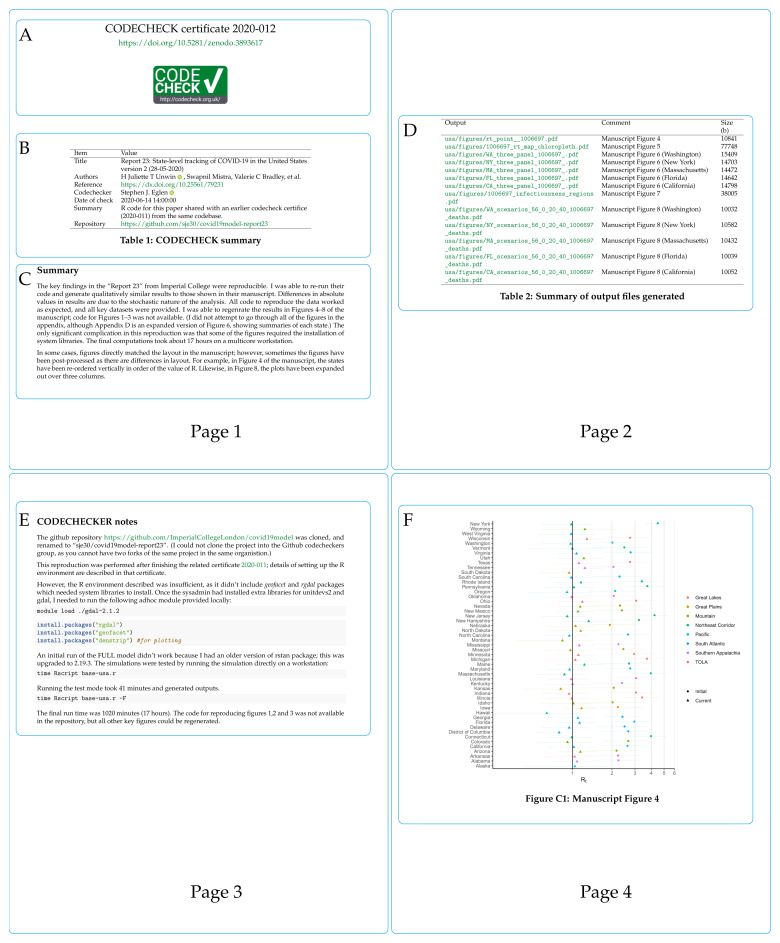
Annotated certificate 2020–012 ^51^ (first four pages only).

Figure 4 shows pages 1–4 (of 10) of an example certificate to check predictions of COVID-19 spread across the USA ^51, 52^. Figure 4A shows the certificate number and its DOI, which points to the certificate and any supplemental files on Zenodo. The CODECHECK logo is added for recognition and to denote successful reproduction. Figure 4B provides the key metadata extracted from codecheck.yml; it names the paper that was checked (title, DOI), the authors, the codechecker, when the check was performed, and where code/data are available. Figure 4C shows a textual summary of how the CODECHECK was performed and key findings. Figure 4D (page 2 of the certificate) shows the outputs that were generated based on the MANIFEST of output files in the CODECHECK. It shows the file name (Output), the description stating to which figure/table each file should be compared in the original paper (Comment), and the file size. Page 3 of the certificate, Figure 4E gives detailed notes from the codechecker, here documenting what steps were needed to run the code and that the code took about 17 hours to complete. Finally, page 4 of the certificate shows the first output generated by the CODECHECK Figure 4F. In this case, the figure matched figure 4 of 52. The remaining pages of the certificate show other outputs and the computing environment in which the certificate itself was created (not shown here).

### Tools and resources

We use freely available infrastructure, GitHub and Zenodo, to run our system. The codecheckers **GitHub** organisation at https://github.com/codecheckers contains projects for managing the project website, the codecheckers community and its discussions, code repositories, and the main register of CODECHECKs. Both the project website https://codecheck.org.uk/ and the register at https://codecheck.org.uk/register are hosted as GitHub pages. The register database is a single table in CSV format that connects the certificate identifier with the repository associated with a CODECHECK. Each of these repositories, which currently can be hosted on GitHub or Open Science Framework, contains the CODECHECK metadata file codecheck.yml. The register further contains a column for the type of check, e.g., community, journal, or conference, and the respective GitHub issue where communications and assignments around a specific check are organised. No information is duplicated between the register and the metadata files. The continuous integration infrastructure of GitHub, GitHub Actions, is used to automate generation of the register. **Zenodo** is our preferred open repository for storing certificates. It mints DOIs for deposits and ensures long-term availability of all digital artefacts related to the project. The CODECHECK community on Zenodo is available at https://zenodo.org/communities/codecheck/. It holds certificates, the regularly archived register ^78^, and other material related to CODECHECK.

A **custom R package**, codecheck, automates repetitive tasks around authoring certificates and managing the register. The package is published at https://github.com/codecheckers/codecheck under MIT license ^79^. It includes scripts to deposit certificates and related files to Zenodo using the R package zen4R ^80^ and for the register update process outlined above. Codecheckers can ignore this package, and use their own tools for creating and depositing the certificate. This flexibility accommodates different skill sets and unforeseen technical advances or challenges.

These tools and resources demonstrate that a CODECHECK workflow can be managed on freely available platforms. Automation of some aspects may improve turnaround time. Our main resource requirements are the **humans** needed for managing the project and processes and the codecheckers. All contributions currently rely on (partly grant-based) public funding and volunteering.

## Related work

The journal *ACM Transactions on Mathematical Software (TOMS)* recently established a “Replicated Computational Results” (RCR) review process ^81^, where “replicable” is the same as our use of “reproducible”. Fifteen RCR Reports have been published so far (search on https://search.crossref.org/ with the term " Replicated Computations Results (RCR) Report" on 2020-12-10). and the process is being extended extended to the ACM journal *Transactions on Modeling and Computer Simulation*. The TOMS RCR follows CODECHECK principles 1–4, although our work was independently developed of theirs. The TOMS editorial ^81^ shares similar concerns about selection of reviewers, as we discussed above. Unlike existing CODECHECK certificates, the RCR reports undergo editorial review. Publication of the RCR report recognises the efforts of the reproducing person, while the potential for this motive to be a conflict of interest is acknowledged. TOMS also recognises reviewer activity in a partnership with Publons (see https://authors.acm.org/author-services/publons). As well as this, ACM provides several badges to indicate what kind of artifact review or reproduction a paper submitted to an ACM journal completed ( https://www.acm.org/publications/policies/artifact-review-and-badging-current), but does not provide nor require a specific review process. In principle, these badges could be awarded by a codechecker, too, though the different levels and even partial replacement of artifacts required to achieve a *Results Reproduced* go beyond a CODECHECK’s scope. A completed check certainly warrants the ACM badge *Artifacts Evaluated - Functional* and possibly *Artifacts Evaluated - Reusable* and likely *Artifacts Available*, depending on additional requirements by implementing journals. However, we do not require codecheckers to evaluate code quality or ensuring proper archival of artifacts though, in our experience, they are likely to encounter or comment on these topics. This activity in the ACM journals can be seen as one possible process within a CODECHECK system, and clearly shares much in spirit. CODECHECK, however, specifically aims to give codecheckers recognition as reviewers. In our view, the reviewer role removes the possible conflict of interest while keeping the public acknowledgement. Specific to the field of mathematics, the RCR is also expected to apply a review of the software itself if the system it runs on cannot be evaluated by an independent party. The TOMS RCR creators concur with the importance of communication, expect collaboration between author and RCR reviewers, share the considerations around reviewer selection, and also put trust in reviewer judgement over numerical bit-wise perfection. A key difference is that for TOMS RCR, authors opt-in with an *RCR Review Request* and the RCR reports are published in the TOMS journal next to the actual papers.

Several journals provide special article types for reproductions of published papers. *Information Systems* has an invitation only Reproducibility Section for articles describing the reproducibility efforts of published articles, which are co-authored by the original authors and the reproducibility reviewer(s) (see https://www.elsevier.com/journals/information-systems/0306-4379/guide-for-authors).

*Nature Machine Intelligence* recently introduced a new type of article, the reusability report ^82^. Inspired by the detailed and nuanced submissions to a reproducibility challenge, the reusability report focuses on the exploration of robustness and generalizability of the original paper’s claims ^82^. This answers the specific community’s challenges around computational reproducibility and also values these kinds of contributions as independent publications, which goes beyond the goals of CODECHECK. The journal *Cortex* has a special article type *Verification Reports*, which are actually about replication of results and are very well designed/reasoned ^83^. The *Journal of Water Resources Planning and Management’s* policy recognises reproducible papers in a special collection and incentivises authors with waived or reduced fees ^84^. In a similar vein, the CODECHECK certificates could also be published as a special article type within journals. Finally, the *Journal of Open Source Software* provides its reviewers with a checklist of items to check during review (see https://joss.readthedocs.io/en/latest/review_checklist.html#software-paper), effectively providing a much more detailed form of check for scientific software that could complement CODECHECKs, too.

Going beyond individual articles, the journal *ReScience C* publishes only replications, also requiring open code and replication by a third party. The journal now accepts “Reproduction reports” that describe if some code accompanying a published article can (or can not) reproduce the same results as shown in the article. ReScience C also relies on free infrastructure (GitHub and Zenodo).

For research with high stakes, where reproduction would be too weak and post-publication replication possibly too late because of policy impact, Benjamin-Chung *et al*. ^85^ propose *internal replication*. A computational workflow that has undergone internal replication would likely be of high quality and relatively easy to check. Similarly, internal CODECHECKs may be used, with the same limitations such as group think ^85^, to ensure reproducibility before submission. Such internal checks are professionalised in local reproduction services, such as CISER R-squared or YARD, or in communities such as Oxford’s code review network.

Gavis and Donoho ^86^ propose a new discipline and infrastructure for reproducible computational research. Their specific packaging format, provenance record, and cryptographic *Verifiable Result Identifier* would indeed provide excellent reproducibility. However, the system is also complex and since its creation in 2011 we are not aware of any publisher using it; also, the system is not open source. In comparison, CODECHECK is less powerful but also much more flexible and less dependent on specific tools or infrastructure. If data and code are deposited properly, i.e., very unlikely to disappear, then the certificate’s DOI is practically close to the cryptographic identifier.

Another platform for publishing results of reproductions is *SciGen.Report*. It is a community-run independent platform to foster communication on reproducibility. People can report on fully, partially, or failed reproductions of articles after publication.

CODECHECK is uniquely designed to be adopted across journals or events and to build a community of codecheckers. CODECHECK shares its interdisciplinary nature with other community initiatives concerned with reproducibility awareness, education, and support, such as ReproHack, Code Copilot, or Papers with Code. The latter recently announced a collaboration with the preprint server *arXiv* on providing data and code supplements for machine learning manuscripts and runs a reproducibility challenge. Likewise, different disciplines and journals provide reproducibility checklists, e.g., science and engineering ^87^ or GIScience ^88^, which naturally share some aspects while addressing particularities as well as addressing researchers from different fields. Regarding the education and guidance for authors, we see CODECHECK’s role as referencing and linking educational efforts and helpful material, not as creating and maintaining such content.

## Limitations

**Isn’t CODECHECK what peer review should be doing already?** On the surface, yes, but peer reviewers are overburdened enough and asking them to do more work around peer review is not likely to succeed. When an editor (Tsuyoshi Miyakawa) requested raw data from n=41 authors before reviewing, 21 authors withdrew their manuscripts; 19 of the 20 remaining articles were rejected after peer review ^89^. Such basic checks require effort from editors, yet they only rely on the availability of data files and the content of the paper. These availability checks can be enhanced by having more complex CODECHECKs request the code and then execute it. This might fall within idealistic expectations of peer review, but is rare. Establishing a CODECHECK workflow acknowledges that peer reviewing practices have been unable to adapt to the challenges of computational papers. The concept of a CODECHECK, just as the concepts of reproducible research and Open Science, may be transitional by nature. If the activities described here as being part of a CODECHECK are integrated into the publication process the initiative will have succeeded.

**Should CODECHECK requirements be more demanding?** CODECHECK by design does not require authors to provide (and sustain) an eternally functional computational workflow nor suggests a specific software stack or practical approach. Creating something that anyone can reproduce has been called a fool’s errand and we tend to agree. However, the package of data, code, and documentation collaboratively created by authors and codecheckers is a snapshot of a working analysis that greatly increases the likelihood of a successful reproduction and the possibility that a computational workflow can be extended by third parties in the future, if they have access to suitable resources and matching skill set. The CODECHECK principles help to make very clear what a CODECHECK badge on a paper means and also ensure a minimum standard that other processes or badges may not have, e.g., only superficially checked self-awarded badges ( https://www.cambridge.org/core/journals/environmental-data-science/information/instructions-for-authors). 

 Concrete implementations of CODECHECK workflows, especially for specific disciplines, may reify much more helpful guidelines for authors on how to create reproducibility packages. Our author-friendly “low bar” should not stay low forever, but cultural change takes time and the encouragement and guidance that CODECHECK, as part of the widely accepted peer review concept, can provide may eventually allow the bar to be raised much higher, e.g., with executable research compendia ^90^, “Whole Tales” ^91^, or continuous analysis ^92^. However, considering that missing artefacts and lack of documentation have repeatedly been identified as key barriers to reproducibility (e.g., 29, 93), we would not underestimate the power of a simple check. For example, ModelDB curation policies require that only one figure need be manually reproduced ^94^, but that has not limited the usefulness nor success of the platform.

A codechecker does not fulfil the same role as a *statistical reviewer*, as it is applied by some journals in the biomedical domain (cf. 95, 96). The statistical reviewer evaluates the appropriateness of statistical methods ^96^ and can support topical reviewers if, e.g., complex methods or sophisticated variants of statistical tests are applied ^95^. The codechecker may go equally deep into the review, but only if they have the expertise and time. We can imagine a tiered CODECHECK workflow where a codechecker could, just as a conventional reviewer could, recommend a detailed code review (see next paragraph) to the editor if they come upon certain issues while examining the work.

A codechecker does not conduct a *code review*. Code reviews are valuable to improve reproducibility and reusability, and their proponents even believe they can improve the research ^97^. Code reviews, however, have quite different structural challenges and require even more resources. That said, a well-reviewed codebase is likely to be easier to codecheck, and the awareness of high-quality code raised through CODECHECK may lead to more support for code reviewing. Initiatives and journals that conduct software reviews independent of a specific publication or venue include ROpenSci, PyOpenSci, and JOSS. Furthermore, the codechecker’s task list is intentionally not overloaded with related issues such as ensuring proper citation of data and software or depositing material in suitable repositories. Nevertheless, codecheckers are free to highlight these issues.

**How are failures during checks handled?** We do not yet have a process for denoting if a reproduction fails, as our case-studies were all successful. In the case that a journal adopts CODECHECK for all submissions, the question remains as what to do if a check fails, after exhausting efforts between author and codechecker to reproduce the computational workflow. A negative comment in a CODECHECK certificate or a failed check does not necessarily mean the paper or research is bad (cf. discussion on negative comments in 17). We doubt that publicly reporting failures (i.e., the code would not run) will increase overall reproducibility, and may prohibit authors from sharing their work, which is always more desirable than nothing shared. Therefore, we recommend sharing interim reproduction efforts only with the authors, even if that means that volunteer efforts may go unnoticed if no certificate is published. Rosenthal *et al*. ^98^ discuss such incentives for different actors around the implementation of reproducibility. We see CODECHECK as one way for organisations to invest in reproducibility by creating incentives until reproducible computations become the norm.

**Who will pay for the compute time?** For papers that take significant compute time (days, not minutes), it is unclear who will pay for it. One must carefully consider the sustainability of rerunning computations and the environmental impact large calculations, such as training machine learning models, have. A pragmatic workaround is to request that authors provide a “toy” example, or small dataset that can be quickly analysed to demonstrate that the paper’s workflow runs correctly.

**What about my proprietary software and sensitive data?** Given the prevalence of proprietary software, e.g MATLAB, in some disciplines we pragmatically decided that we should accept code as long as we could find a machine with suitable licences to run it. However, this prohibits us from using open infrastructure for reproducibility (cf. 10, 99) and requires the codechecker to have access to that particular software. Non-open software also considerably hampers reuse, especially by researchers from the global south. Likewise, if a research requires specific hardware, e.g. GPUs, we are reliant on the codechecker having access to similar hardware. Both licenses and costs can be barriers to a CODECHECK, but the focus on the codechecker’s assessment provides options to overcome these barriers if needed. Therefore, allowing proprietary software and specialised hardware are compromises that should be reconsidered. In any case, authors must make such requirements clear and the opportunity to answer them must be documented for codecheckers.

Solutions for proprietary and sensitive data exist. Authors can provide synthetic data (cf. 100), some data can effectively be redacted ^101^, and publishers or independent entities can provide infrastructure for sharing data and computational workflows confidentially ^102^ or with access to derived results but not raw data ^100^, i.e., data enclaves ^103^, or domains of reproducibility ^104^.

**Can’t someone cheat?** Yes. We simply check that the code runs, not that is correct or sound science. This “mechanical” test is indeed a low bar. By having code and data openly deposited, third parties can later examine the code, and we hope that knowing the code will be open ensures that authors will not cheat. It also allows researchers, potentially with new methods, to look for errors. This is more effective than engaging in an arms race on building methods to detect malicious intent now with closed datasets and code. This is analogous to storing blood samples of sport champions today to possibly detect doping in the future with more sensitive methods (cf. 105). Another comparison that helped us define the scope of a CODECHECK is that we think of the codechecker as forensic photographer, capturing details so that an investigator may later scrutinise them.

**Who’s got time for more peer review?** Agree; codechecking takes time that could otherwise be used for traditional peer review. However, a CODECHECK is different from peer review. First, the **technical nature** of a CODECHECK sets clear expectations and thereby time budget compared to conventional peer review. For example, authors are told what to provide and the codechecker can be told when to stop. Codecheckers can always directly ask the author when clarification is required, thereby increasing **efficiency**. Second, the specific skill set of a codechecker allows for **different groups** to participate in the review process. ECRs might be attracted to learn more about recent methods, peer review, and reproducibility practices. Research Software Engineers who might not regularly be involved in writing or reviewing papers might be interested in increasing their connection with scholarly practices. An extra codechecker may simplify the matchmaking an editor does when identifying suitable reviewers for a submission, as technical and topical expertise can be provided by different people (cf. segmentation of multidisciplinary works ^106^). Third, recall that CODECHECKs should always be publicly available, unlike peer review reports. With code and computational workflows, the codechecker’s feedback may directly impact and improve the author’s work. The public certificates and contributions provide **peer recognition** for the codechecker. Fourth, we found that focusing on the computational workflow’s mechanics and interacting with the author makes reproductions **educational**. It also is a different role and, as such, could be a welcome option for researchers to give back their time to the community.

While such benefits are also part of idealistic peer review, they are mostly hidden behind paraphrased anonymous acknowledgement.

**Do computational workflows need to be codechecked multiple times?** If a paper is checked at the start of peer review, it might need re-checking if the paper is modified during peer review. This is inevitable, and happened to us ^51^. This is desirable though, if interactions between author, reviewer, and codechecker led to improvements. Checking the manuscript the second time is likely to be much less work than the first time.

**What does it mean for a figure to be reproducible?** Automatically detecting if a codechecker’s results are “the same” as an author’s is more challenging than it might appear. That is why we do not require results to be *identical* for a CODECHECK to pass but simply that the code runs and generates output files that the author claims. Stochastic simulations mean that often we will get different results, and even the same versions of libraries can generate outputs that differ by operating system ^107^. While reproducibility practices can mitigate some of these problems, e.g., by using a seed, the flexibility of the human judgement is still needed, rather than bitwise reproducibility. The codechecker is free to comment on visible differences in outputs in their report.

**Shouldn’t the next step be more revolutionary?** CODECHECK’s approach is to acknowledge shortcomings around computational reproducibility and to iteratively improve the current system. It remains to be proven whether this approach is welcomed broadly and if involving publishing stakeholders helps to further the cause. We have discussed more stringent rules at length, e.g. only considering fully free and open source software, diamond Open Access journals, but we eventually decided against them on the level of the principles. For the CODECHECK community workflow, documented at https://codecheck.org.uk/guide/community-process, and the volunteer codechecker community, these requirements can be reconsidered.

We have deliberated requiring modern technologies to support reproducibility (cf. 10), focusing instead on the human interface and the judgement of experienced researchers and developers as a more sustainable and flexible approach. All types of research can adopt CODECHECK due to its flexible design. CODECHECK could include automated scoring (e.g., 108), yet automation and metrics bear new risks. The focus of the CODECHECK principles on code execution allows journals and publishers to innovate on financial models and peer review practices at their own pace.

## Conclusions and future work

CODECHECK works — we have reproduced a considerable number of computational workflows across multiple disciplines, software stacks, and review processes, and we have documented all results transparently in CODECHECK certificates. The creation of certificates and interactions with authors and editors shaped the principles and the CODECHECK workflow and also confirmed the approach taken. This result corroborates findings from similar evaluations of reproducible computational research in journals and conferences. CODECHECKs increase transparency of the checked papers and can contribute to building trust in research findings. The set of shared principles and common name, through recognition value, will allow researchers to judge the level of scrutiny that results have faced. CODECHECK requires direct acknowledgement of the codechecker’s contributions, not indirectly via citations of reproductions or informal credit.

CODECHECK however harbours the same limitations as peer review in general and is closely connected to larger disruptions and challenges in scholarly communication ^7, 109, 110^, including the tensions between commercial publishing and reviewers’ often free labour, and a global pandemic that has jumbled up academic publishing and exposed a broader general audience to preprints ^111^. Establishing CODECHECK workflows must be seen as interconnected with much larger issues in research, such as broken metrics or malpractice triggered by publication pressure ^112, 113^. We certainly do not want the binary attribute of “code works” to become a factor in bibliometric approaches for performance assessments.

While developed for the current “paper”-centric publication process, the CODECHECK principles would also work well with novel publication paradigms, e.g., peer-reviewed computational notebooks ^114^, iterative and granular communication of research outputs, articles with live-code ^115^ such as eLife’s ERA, decentralized infrastructure and public reviewer reputation systems ^116^, and completely new visions for scholarly communication and peer review, such as described by Amy J. Ko in *A modern vision for peer review*. A CODECHECK’s impact on the published research outputs and the required infrastructure would also support answering needs for better integration of research outputs and more openness ^117^. An explicit segmentation of research steps could even make the focus of a CODECHECK easier by only checking the “analysis” sub-publication. The discovery of CODECHECKs could be increased by depositing certificates into public databases of reproductions, such as *SciGen.Report*. Public researcher profiles, such as ORCID, may consider different types of reviewer activity to capture how independent code execution contributes to science. Notably, the discussed limitations are largely self-imposed for easier acceptance and evolutionary integration, as to not break the current system and increase demands gradually without leaving practitioners behind. A CODECHECK system, even if temporarily adopted as a sustainable transition towards more open publication and review practices, can contribute to increased trust in research outputs. Introducing CODECHECK should be informed by lessons learned from (introducing) open peer review ^15^. Our conversations with publishers and editors indicate a willingness to adopt open practices like these, but that it is hard to innovate with legacy infrastructure and established practices.

More reproducible practices initiated by CODECHECKs could lead communities to reach a state where authors provide sufficient material and reviewers have acquired sufficient skills that peer reviewers will generally conduct a CODECHECK-level of checking; only in especially sophisticated cases will a specialised codechecker be needed. The main challenge for us remains getting journals to embrace the idea behind CODECHECK and to realise processes that conform to the principles, whether or not they use CODECHECK by name. We would be keen to use the flexibility of the principles and cooperate with journals to learn more about the advantages and yet unclear specific challenges – e.g do CODECHECKs really work better with open peer review? To facilitate the adoption, the CODECHECK badge is, intentionally, not branded beyond the checkmark and green colour and simply states “code works”.

Future CODECHECK versions may be accompanied by studies to ensure codechecking does not fall into the same traps as peer review did ^16^ and to ensure positive change within the review system. This *cultural change*, however, is needed for the valuation of the efforts that go into proper evaluation of papers. Journals can help us to answer open questions in our system: What are crucial decisions or pain points? Can authors retract code/data once a CODECHECK has started? What variants of CODECHECKs will be most common? How will open CODECHECKs influence or codevelop with the scope and anonymity of conventional review over time?

The question of training codecheckers is also relevant. We expect a mentoring scheme within the CODECHECK community, in which experienced codecheckers will provide on-the-job training or serve as fallback advisors, would be most suitable. Given the difficulty to document solutions for the unique problems every check has, practical experience in the craft of codechecking is paramount. Codecheckers may also be found by collaborating with reproducible research initiatives such as ReproHack, ReproducibiliTea, ^118^, and Repro4Everyone ^119^. The initial reaction of researchers to these ideas shows that scholarly peer review should continue on the path towards facilitating sharing and execution of computational workflows. It is perhaps too soon to see if CODECHECK increases reuse of code and data, and we would certainly value a longer-term critical assessment of the impact of material that has been checked.

## Data availability

Zenodo: codecheckers/register: CODECHECK Register Deposit January 2021 http://doi.org/10.5281/zenodo.4486559 ^117^.

This project contains the following underlying data:

- register.csv. List of all CODECHECK certificates with references to repositories and reports.

Data are available under the terms of the Creative Commons Attribution Share Alike license (CC-BY-SA 4.0 International).

## Software availability

Codecheckers GitHub organisation: https://github.com/codecheckers

CODECHECK community on Zenodo: https://zenodo.org/communities/codecheck

codecheck R package: https://github.com/codecheckers/codecheck

Archived R package as at time of publication: http://doi.org/10.5281/zenodo.4522507 ^79^

License: MIT
