# Supplementary material for: CODECHECK: an Open Science initiative for the independent execution of computations underlying research articles during peer review to improve reproducibility
Source: F1000Res. 2021 Jul 20;10:253. Originally published 2021 Mar 30. [Version 2] doi: 10.12688/f1000research.51738.2 (PMC8311796; doi:10.12688/f1000research.51738.2)
Supplement: Peer review history

# Peer review: Reviewer response for version 2

**DOI:** 10.5256/f1000research.57857.r89926

**Published:** 2021-07-20

**Authors:** Nicolas P. Rougier

**Affiliations:** 1 Inria Bordeaux Sud-Ouest, Talence, France

Thank you for all your answers. I now realize that some of my questions we already addressed in the manuscript but for some reason I overlooked them. Sorry for that. I'm satisfied with the new version and the answer to my questions.

Is the rationale for developing the new method (or application) clearly explained?

Yes

Is the description of the method technically sound?

Partly

Are the conclusions about the method and its performance adequately supported by the findings presented in the article?

Yes

If any results are presented, are all the source data underlying the results available to ensure full reproducibility?

Yes

Are sufficient details provided to allow replication of the method development and its use by others?

Yes

Reviewer Expertise:

Computational  Neuroscience, Open Science

I confirm that I have read this submission and believe that I have an appropriate level of expertise to confirm that it is of an acceptable scientific standard.

# Peer review: Reviewer response for version 1

**DOI:** 10.5256/f1000research.54932.r82472

**Published:** 2021-04-21

**Authors:** Sarah Gibson

**Affiliations:** 1 The Alan Turing Institute, London, UK

**Paper Summary**

 This paper outlines a set of principles and a community of practice for verifying computational analyses can be run and research artefacts reproduced as part of, or in addition to, traditional peer review processes. The ongoing scientific reproducibility crisis and current lack of many (or any) standards for checking computational research in the publishing industry makes this an important, new framework to share with the community.

 The authors demonstrate a deep and thoughtful knowledge of the cultural barriers surrounding such technological checks for peer review, such as time, expertise, and bitwise comparative reproducibility. They acknowledge that the specific incarnation of the CODECHECK practice outlined in this paper is limited to provide a low barrier for entry in order to encourage adoption, but do detail the scope in which such a workflow could be adapted and built upon to raise that bar and perform more stringent checks. Specifically, the principles are not technology-based to allow for flexibility in the complexity and domain of computational research to be checked. I particularly appreciated the authors’ recommendation/suggestion that CODECHECKs become a platform for engaging Early Career Researchers in the peer review process.

 Alongside CODECHECK’s own workflows (which are openly published on GitHub and Zenodo), the paper outlines many similar and related initiatives that fall within the CODECHECK framework providing a wealth of examples for the community to draw inspiration from when designing and applying their own CODECHECK workflows.

**Is the rationale for developing a new method clearly explained?**

 The authors show a deep knowledge of the pitfalls of traditional peer review of static research artefacts and clearly identify and outline the rationale for a peer review-like system capable of assessing computation-based research.

**Is the description of the method technically sound?**

 I’m going to answer a slightly different question of “Is the description of the method *culturally* sound?” This is because the authors have intentionally not provided a technological methodology for completing a CODECHECK so as to avoid vendor lock-in (e.g. cloud platform providers) and to provide flexibility for applying the methodology to a range of computational research domains. Instead, the focus of the methodology is on building a community of practice around having code mechanically checked by someone with comparable technical expertise from outside the project.

 The authors demonstrate a considerate knowledge of the burden of verifying computational reproducibility on both authors and peer reviewers and aim, not to increase this burden, but to provide an entry point into a world where checking research code can be run and produces the artefacts as they are presented in the paper is normalised. I think their recommended approach focussing on communication between codecheckers and authors, codecheckers will check and not fix, and codecheckers being an additional role to the traditional peer reviewer will aid early adoption of this framework.

**Are sufficient details provided to allow replication of the method development and its use by others?**

 The concept of CODECHECK is intentionally presented as a set of principles and example workflows, as opposed to fixed, step-by-step actions, to allow for flexibility across computational complexity and research domains. The principles, example workflow, and potential variations under this framework are explained in depth and examples of workflows that fall under the CODECHECK framework from other publishers and/or conferences are provided, alongside CODECHECK’s own community. From this wealth of detail, I believe that others would be able to replicate, adapt and apply a CODECHECK-like workflow in their journal or community.

**Are the conclusions about the method and its performance adequately supported by the findings presented in this article?**

 It is encouraging to see that the community feedback from authors and publishers shaped the workflow and principles that uphold CODECHECK and a number of certificates have already been issued under this framework. This shows that the workflow of a CODECHECK as outlined in the paper is achievable in partnership with current peer review operations. However, I would like to see the impact of the CODECHECK certificates issued. Is there any community feedback on the transparency and reusability of research published with CODECHECK certificates? This is perhaps too big of an ask this early in the initiative as research reuse and citations are independent factors of the publication and peer review of this specific paper - but I’d still be interested in any insights the authors have to offer on this topic.

Is the rationale for developing the new method (or application) clearly explained?

Yes

Is the description of the method technically sound?

Yes

Are the conclusions about the method and its performance adequately supported by the findings presented in the article?

Partly

If any results are presented, are all the source data underlying the results available to ensure full reproducibility?

No source data required

Are sufficient details provided to allow replication of the method development and its use by others?

Yes

Reviewer Expertise:

As a Research Software Engineer, I don't have a specific area of research any more. I have skills and expertise in software best practices, computational reproducibility and cloud computing infrastructure, which I have gained through the open source communities Project Binder (running mybinder.org) and The Turing Way (a pedagogical resource which includes a volume on reproducibility) alongside working on a range of projects within the Alan Turing Institute.

I confirm that I have read this submission and believe that I have an appropriate level of expertise to confirm that it is of an acceptable scientific standard.

# Response

**Published:** 2021-06-16

**Authors:** Stephen Stephen

**Affiliations:** University of Cambridge, UK

> 1. Is there any community feedback on the transparency and reusability of research published with CODECHECK certificates?

 This is an excellent question.  It is perhaps too early for us to assess this given most certificates are under a year old, but we are not aware of any reuse yet of the codecheck-deposited material.  However, we certainly think it interesting to try and monitor this over a longer (3-5 year) timescale if possible.  As well as looking for citations of certificates, we could also check for forks of our repositories, and download statistics from Zenodo.  We note this as a good closing point for our article.

# Peer review: Reviewer response for version 1

**DOI:** 10.5256/f1000research.54932.r82470

**Published:** 2021-04-19

**Authors:** Nicolas P. Rougier

**Affiliations:** 1 Inria Bordeaux Sud-Ouest, Talence, France

In this article, authors propose to implement a procedure to check for the code accompanying a submission to a journal. To do so, they describe a pipeline made of 6 steps that ultimately lead to the delivery of a code check certificate meaning that someone external to the author's lab has managed to re-run the code. At this point, no checking that the results are correct is necessary. The authors already

 issued several codecheck certificates in different disciplines. I find the idea really nice and certainly necessary but I've a few questions (even though some of them are already addressed in the "limitations" section). Given the structure of the paper, I'll just list my questions here:

- How does CODECHECK compare to ACM Artifact reviews badges? (https://www.acm.org/publications/policies/artifact-review-and-badging-current)
- What would be the incentive for someone to code check the code? Being aware of the increasing difficulty in finding reviewers, I don't think it would be easy to recruit people to perform a task that can rapidly become very technical and time consuming.
- How do you handle the case when specific hardware is necessary (e.g. NVidia GPU)? Is it documented somewhere such that code-checkers might first verify if they have the necessary hardware to run the code?
- How do you establish a check has failed? For example, what happens if a code-checker gets a segfault (for some unknown reason) and the author is unable to help. Is it deemed failed?
- Who will pay for the computing resources needed to run heavy simulations and/or to acquire necessary software such as e.g. Matlab? When a simulation consumes a lot of resources, it might wise to give the checker access to computing resources. This could be paid for by the journal.
- I did not see in the report example a description of the environment necessary to run the software. How did you solve the "dependency hell"? Since the code might break at some point in the future because of incompatibility in some libraries or environments, it would be necessary to have a mechanism describing the running environment such that it can be re-run later.
- What do you recommend if the reviews are both excellents but the code check failed? Does this mean the paper is blocked until code check passes or rejected or else?
- The code check proposal is close to some extents to the Journal of Open Science Software where each reviewer is assigned a list of things to check during the review. Do authors consider this pipeline when establishing their own pipeline?
- To what extent the codecheck certificate can be updated automatically via some kind of "manual continuous-integration"? I mean that when reading a paper online, would it be possible to click a button to test if the code still runs considering the latest versions of libraries? (for example, the certificate has been issued for Python 2 but I want to know if this is usable with Python 3).
- When you look at journals advertising open data policies, it is unfortunately not rare to find articles in these same journals without the actual data. Do you have some suggestion for educating editors to actually enforce the code check a journal adopt it?

    

 Some suggestions:

- The badge that is delivered would need some time information since the check is valid at one point in time (with a given software stack) and does not guarantee future runs.
- For specialized journals, you could consider to offer a common generic environment where a code could be first tested. It this fails, then you would need only to slightly modify the environment to add missing dependencies. For example, in neuroscience, a Neuro Debian would probably suit the needs of a large number of models.
- As editor-in-chief of ReScience C, I would like to inform authors that the journal now accepts "reproduction report". The idea it to try to re-run the code accompanying a published article and to report if it succeeded or failed. Our own procedure to check for reproduction is not standardized and we'll certainly benefit from the code check initiative.

Overall, it's nice to have a clean description of a pipeline to check for code even though some questions need to be addressed. Also, I'm not too confident that journals will adopt it immediately and I'm afraid such initiative will take time to be generalized. But we have to start somewhere.

Is the rationale for developing the new method (or application) clearly explained?

Yes

Is the description of the method technically sound?

Partly

Are the conclusions about the method and its performance adequately supported by the findings presented in the article?

Yes

If any results are presented, are all the source data underlying the results available to ensure full reproducibility?

Yes

Are sufficient details provided to allow replication of the method development and its use by others?

Yes

Reviewer Expertise:

Computational  Neuroscience, Open Science

I confirm that I have read this submission and believe that I have an appropriate level of expertise to confirm that it is of an acceptable scientific standard, however I have significant reservations, as outlined above.

# Response

**Published:** 2021-06-16

**Authors:** Stephen Stephen

**Affiliations:** University of Cambridge, UK

> 1. How does CODECHECK compare to ACM Artifact reviews badges?

 > (https://www.acm.org/publications/policies/artifact-review-and-badging-current)

 These badges, introduced in August 2020, show whether code is available (different levels) and reproduces same results.  In principle CODECHECKER could award these badges (artifacts evaluated, functional).  We have made a note to this effect in the manuscript (Related work, end of paragraph 1.)

 > 2. What would be the incentive for someone to code check the code?

 > Being aware of the increasing difficulty in finding reviewers, I

 > don't think it would be easy to recruit people to perform a task

 > that can rapidly become very technical and time consuming.

 This was addressed in our section "Who's got time for more peer review?" We would however note that we have a pool of about 20 volunteers currently willing to do codechecks.

 > 3. How do you handle the case when specific hardware is necessary

 > (e.g. NVidia GPU)? Is it documented somewhere such that

 > code-checkers might first verify if they have the necessary hardware

 > to run the code?

 This was handled in the limitation "What about my proprietary software and sensitive data." but we now mention hardware too in the first paragraph of that section.

 > 4. How do you establish a check has failed? For example, what

 > happens if a code-checker gets a segfault (for some unknown reason)

 > and the author is unable to help. Is it deemed failed?

 We hope that codechecker and author can resolve problems, but in the end there may be problems that cannot be solved.  Open infrastructure could help as both author and codechecker can work together in the same environment to minimise these failures.  Ultimately however, there may be failures, which are noted in the section "How are failures during checks handled?".

 > 5. Who will pay for the computing resources needed to run heavy

 > simulations and/or to acquire necessary software such as

 > e.g. Matlab?  When a simulation consumes a lot of resources, it

 > might wise to give the checker access to computing resources. This

 > could be paid for by the journal.

 In the section "Who will pay for compute time?" we mention this problem, and that toy examples might alleviate the need to re-run resource-intensive computations.  We agree that one model might be that a journal provide some resource for this service.  Likewise, in the following paragraph, we describe that our pragmatic approach for now is to find codecheckers that have access to particular software, e.g. MATLAB.

 > 6. I did not see in the report example a description of the

 > environment necessary to run the software. How did you solve the

 > "dependency hell"? Since the code might break at some point in the

 > future because of incompatibility in some libraries or environments,

 > it would be necessary to have a mechanism describing the running

 > environment such that it can be re-run later.

 The short answer is "we didn't".  In the paragraph "Should CODECHECK requirements be more demanding?" we note our low bar of simply getting a codecheck to run once.  We do, however, encourage CODECHECKERS to describe the environment in free text form in their report.  Moving towards machine-readable descriptions would be a natural extension.

 > 7. What do you recommend if the reviews are both excellents but the code

 > check failed? Does this mean the paper is blocked until code check

 > passes or rejected or else?

 This is up to the editor of the journal -- see the "Importance" dimension of Figure 3.  At one end, it could indeed be a "strict requirement" to get a codecheck certificate for the paper to be accepted.  On the other hand, it could be entirely optional.

 > 8. The code check proposal is close to some extents to the Journal

 > of Open Science Software where each reviewer is assigned a list of

 > things to check during the review. Do authors consider this pipeline

 > when establishing their own pipeline?

 We have not considered this pipeline, nor do we have an explicit idea.  We now note this reviewer list at the end of the third paragraph of "Related Work".

    

 > 9. To what extent the codecheck certificate can be updated

 > automatically via some kind of "manual continuous-integration"? I

 > mean that when reading a paper online, would it be possible to click

 > a button to test if the code still runs considering the latest

 > versions of libraries?  (for example, the certificate has been

 > issued for Python 2 but I want to know if this is usable with Python

 > 3).

 To follow on from point 6, this would make a natural extension, but for now we are still considering one point in time, and keeping the requirements as close to the authors as we can.

 > 10. When you look at journals advertising open data policies, it is

 > unfortunately not rare to find articles in these same journals

 > without the actual data. Do you have some suggestion for educating

 > editors to actually enforce the code check a journal adopt it?

 We share this concern, and unfortunately have no simple suggestions for helping editors.  At this early stage, we think the approach should be one of encouraging uptake, rather than mandating it.  We also hope that having specific in-house experience, e.g. editorial staff to examine for code and data availability, can note this.  But at the end of the day, this again is dependent on the journal's workflow.

     

 > 11. The badge that is delivered would need some time information

 > since the check is valid at one point in time (with a given software

 > stack) and does not guarantee future runs.

 Great idea. we could add the certificate number to the URL, or add the certificate number.  We will try to implement this when revising our workflows.  Nevertheless, the point in time and software stack should be documented via the certificate already now.

 > 12. For specialized journals, you could consider to offer a common

 > generic environment where a code could be first tested. It this

 > fails, then you would need only to slightly modify the environment

 > to add missing dependencies. For example, in neuroscience, a Neuro

 > Debian would probably suit the needs of a large number of models.

 Yes. We will certainly bear this in mind in future work, especially for author guidelines.

 > 13. As editor-in-chief of ReScience C, I would like to inform

 > authors that the journal now accepts "reproduction report". The idea

 > it to try to re-run the code accompanying a published article and to

 > report if it succeeded or failed. Our own procedure to check for

 > reproduction is not standardized and we'll certainly benefit from

 > the code check initiative.

 Thank you for noting this.  We now mention the reproduction report in the manuscript where we describe Rescience C.

 > Overall, it's nice to have a clean description of a pipeline to

 > check for code even though some questions need to be

 > addressed. Also, I'm not too confident that journals will adopt it

 > immediately and I'm afraid such initiative will take time to be

 > generalized. But we have to start somewhere.

 We share your realistic assessment that (a) journals may be slow to adopt but that (b) we should start somewhere.
